# Square peg round hole: navigating operational and interdisciplinary challenges in a co-produced, community-based research partnership

**DOI:** 10.1186/s40900-025-00769-1

**Published:** 2025-08-18

**Authors:** Emma Tuschick, Jo Smith, Matthew Cotton, Jibin He, Ashley Blacklock, Cara Jordan, Helen J Moore, Amelia A Lake, Lisa Harris, Lesley Lowe, Kim Baines, Joe Dunne, Emma L. Giles

**Affiliations:** 1 School of Health and Life Sciences, Teesside University, Middlesbrough, TS1 3BX UK; 2 Research and Development Team, Flatts Lane Centre, Tees, Esk and Wear Valleys NHS Foundation Trust, Flatts Lane, TS6 0SZ Normanby, Middlesbrough, UK; 3 School of Social Sciences, Humanities and Law, Teesside University, Middlesbrough, TS1 3BA UK; 4 The Centre for Translational Research in Public Health, Fuse, Newcastle, UK; 5 FareShare North East, Newcastle upon Tyne, UK; 6 Middlesbrough Environment City, Middlesbrough, TS5 7YN UK

**Keywords:** Co-creation, Communication, Collaboration, Opportunities, Challenges

## Abstract

**Background:**

Inclusive, community-based research is increasingly important for aligning academic work with real-world needs. This paper describes the challenges and successes of a research collaboration between academic, health service, and voluntary sector partners, working together to address nutrition and food waste for individuals with severe mental illness.

**Methods:**

This reflective article reports a primary study evaluation of the process of a co-produced project aimed at developing a nutritious ready meal from surplus food to be distributed through Eco Shops, involving individuals with severe mental illness (SMI). A collaborative partnership was formed between a University, an NHS Foundation Trust, and a Voluntary Sector Organisation in the North-East of England. Data for this process evaluation were collected through a team questionnaire evaluating all aspects of the collaboration, including ethics, governance, and communication practices.

**Results:**

While the overall project achieved positive outcomes, the collaborative research process included substantial challenges in ethical and governance alignment, collaborative working practices, and communication across diverse organisational cultures.

**Conclusion:**

This study highlights the need for flexible governance, clear communication protocols, and effective workload management in cross-sector collaborations. Insights emphasise adapting project frameworks to support interdisciplinary, community-focused research for successful, sustainable partnerships.

**Supplementary Information:**

The online version contains supplementary material available at 10.1186/s40900-025-00769-1.

## Abstract (plain language summary)

This article presents a reflection on how a project in the UK, designed to help reduce food waste in a local community and improve nutrition of local people who have severe mental illness (SMI) trying the free ready meal, was developed and delivered. While the overall project had its own aims, this article focuses specifically on how the collaboration worked in practice.

The project collected feedback from everyone involved using a questionnaire, which looked at how well the partnership worked, including ethics, rules, and communication.

While the project had some good results, it also faced problems. These included difficulties with agreeing on rules, working together, and communicating between different groups. The questionnaire showed that while some parts of the project were successful, there were also delays and misunderstandings because the organisations worked in different ways.

The study shows that for projects like this to work well, it is important to have clear rules, good communication, and a way to manage workload. It also suggests that project plans should be flexible, so they can work well for everyone involved and be successful in the long term.

**Supplementary Information:**

The online version contains supplementary material available at 10.1186/s40900-025-00769-1.

## Background

The concept of research co-creation and co-production has been applied in various forms across disciplines, including healthcare, for several decades [1, 2]. In recent years, large funding bodies, such as the National Institute for Health and Care Research (NIHR) [3] have increasingly recognised the value of involving non-traditional research organisations in health and healthcare research. This trend reflects a broader shift towards more inclusive and participatory research practices, which are seen as essential for ensuring that research is more responsive to the needs of diverse populations [4, 5]. In healthcare research, the meaningful involvement of patients, individuals with lived experience, and service users is increasingly recognised as not only beneficial but essential for ensuring that research outputs are relevant, effective, and meet the needs of those they are intended to benefit [6, 7]. This approach aligns with a broader shift towards a community health approach, and a renewed focus upon participatory, action research, and patient-centred research paradigms, which emphasise the importance of co-producing knowledge with those who have direct experience of the issues being studied [4, 8]. Such engagement helps to bridge the gap between academic research and practical application, thereby enhancing the relevance and impact of research findings. However, the infrastructure, culture, and processes within research institutions do not always keep pace with these advances in research philosophy and design, leading to challenges in implementation [9].

The complexity of healthcare systems further highlights the importance of participatory research, especially in community settings where care is managed primarily on an outpatient basis. In these contexts, the challenges of delivering integrated and coordinated care are heightened by the need to navigate the interdependencies between various healthcare providers, social services, and community-based organisations [10, 11]. Research conducted within these complex environments involves collaboration across multiple organisations. Therefore, the intricacies of aligning different organisational cultures, priorities, and operational processes become even more pronounced [12].

This commentary focuses on a research project that exemplifies such complexities in the UK context, involving three partner organisations: a University, an NHS Mental Health Foundation Trust, and a Voluntary and Community and Social Enterprise (VCSE), all in the North-East of England. These organisations, coming together for the first time as a research partnership, aimed to develop an innovative solution to the twin challenges of improving the social wellbeing and access to nutritious food and reducing edible surplus food waste for people living with severe mental illness (hereafter SMI). Working with people living with SMI, the partnership co-designed, developed, and evaluated a new healthy, affordable ready meal made from surplus food. For this project, we define surplus food as food ingredients and products that are still suitable to eat and use as ingredients but were removed from the commercial food supply chain due to aesthetic criteria or lack of demand [13].

Funded as an exploratory developmental research project by the NIHR [14], the project was designed within a co-production framework, where meal ideas were co-produced by people with SMI recruited via the mental health NHSTrust. These recipe ideas were then cooked and evaluated in collaboration with two Healthy Cooking Advisors from the VCSE organisation. The final product and its package design were produced in the University’s food processing laboratory, with the final product distributed through “Eco Shops”– a local social supermarket network designed specifically to alleviate food insecurity across the Tees Valley region in Northeast England. Social supermarkets operate by offering surplus or donated food at significantly reduced prices, ensuring that those facing financial hardship can access nutritious and affordable groceries [15, 16]. These shops not only help to reduce food waste by redistributing edible surplus from producers and retailers but also foster community support and resilience by addressing the root causes of food insecurity in a dignified and inclusive manner. Thirty ready meals were distributed through three Eco Shops, demonstrating the potential of cross-sector collaboration for social and industrial innovation in addressing health inequalities, wellbeing and food sustainability challenges. The NIHR suggests a focus on getting both the “right people” and the “right processes” in place in the design of collaborative and co-produced research projects. The NIHR recently established a set of guiding principles for engagement research [17] to this effect. They argue that coproduced and collaborative research involves engagement with public and stakeholder representatives in all aspects of the process– research design, case study selection, methodology and process design, data collection, analysis, and process evaluation. Establishing effective collaborative partnerships to achieve these goals is seen as a best practice model in a range of public health settings, particularly where research is designed to address specific user needs. Established principles of coproduction research include the sharing of power between researchers, users and other embedded stakeholder groups, the inclusion of different perspectives, skills and epistemologies, respect for and valuing of diverse knowledge, processes that establish reciprocity, and mechanisms to build and maintain collaborative working practices.

Collaborative models in the field of health research have emerged in response to the challenges of shared partnership working and knowledge coproduction. In particular the *collective impact model* (CIM), designed to promote the “scaling up” of health outcome change [18]. The CIM has five core tenets for knowledge coproduction within a partnership: (1) a shared agenda, (2) shared measurement systems, (3) mutually reinforcing activities, (4) continuous communication, and (5) a central infrastructure. Research into the CIM, when applied to health research partnerships in the UK, demonstrates that it is a generally effective approach to fostering quality improvement initiatives within healthcare settings by promoting structured, cross-organisational collaboration. Evidence from NHS-based and other healthcare setting studies suggests that collaboratives such as those using this type of approach can enhance professional motivation, facilitate shared learning, and lead to measurable improvements in clinical processes and service delivery [19, 20]. The key enablers to the success of the CIM include the presence of local “champions” within a research partnership, establishing a clearly defined common agenda early on within the research process. Barriers include inconsistent leadership engagement, organisational inertia in adopting new coproduced ways of working, and the complexity of the CIM approach [21]. Although the CIM has shown success in changing professional behaviours and improving care processes within UK and other healthcare settings [22], impact on patient outcomes is less consistently evidenced [23], and applications to wider partnership working in public and community health settings is limited [24]. Overall, the model’s strength lies in its ability to align diverse stakeholders around shared goals, but its effectiveness is contingent upon sustained commitment, robust infrastructure, and the capacity to navigate complex inter-organisational dynamics, all of which raise key challenges for NIHR funded projects within University-Primary Care Trust-Community partner research settings.

This article focuses on the challenges and learning gained from co-producing research across three very different partner institutions and with potentially vulnerable people. Our aim is to offer valuable insights for stakeholders involved in cross-sector research, including practical recommendations to inform preparation, governance, timelines, and collaboration. By identifying barriers and proposing realistic strategies, this paper contributes to improving the design and delivery of complex, collaborative research projects. Collaborative research projects involving diverse stakeholders, such as universities, healthcare institutions, and community-based organisations, are becoming increasingly common due to their potential to address complex social and health issues. For meaningful progress, especially in the context of mental health, these partnerships are vital to bridge gaps between inpatient, outpatient, and community-based services. This study highlights how collaborative, co-produced research initiatives can foster continuity across these services, offering a more integrated approach that brings research closer to practical applications. However, despite the growing recognition of the value of such partnerships, these projects frequently encounter significant challenges that can undermine their success. Shared partnership projects often involve navigating divergent organisational cultures, governance structures, and operational procedures, all of which contribute to delays, inefficiencies, and miscommunication. These barriers are not unique to a single project but represent widespread issues inherent in multi-organisational collaborations. By identifying these challenges and offering practical solutions, this paper aims to provide readers with the tools to better manage future collaborations and avoid common pitfalls.

## Method

The reflections in this article are drawn from two primary data sources: a shared team document used throughout the project to log observations, and a questionnaire distributed to all team members toward the end of the project. The questionnaire contained both quantitative (Likert-scale) and qualitative (free-text) questions and asked participants to reflect on various aspects of the research process, including the effectiveness of collaborative practices; clarity of communication; and challenges faced in navigating ethical and governance issues. Participants also highlighted both the strengths and areas for improvement within the partnership, offering valuable lessons for future co-produced research endeavours. The questionnaire was developed collaboratively by the project leads and sent to all 12 project members; 9 responses were received.

Responses were analysed thematically to identify common themes, which informed the structure of this article. In practice we employed a reflexive thematic analytical approach to qualitative analysis. The approach was principally ‘bottom up’ and iterative in the development of themes, drawing thematic categories by following a seven-phase approach building on Braun and Clarke [25]: i.e. familiarisation with the data, generating initial codes, searching for themes, reviewing themes, defining and naming themes, producing a report for researcher review and comment, and then refining and reporting in the final manuscript. One researcher led the initial coding using Nvivo software to develop a ‘top level’ coding framework and starting the theme development. This was then independently reviewed through comparison of coding practice with two additional team members to ensure consistency and depth of interpretation. Discrepancies were discussed collaboratively, and themes were refined through iterative dialogue during a series of in-person meetings. To enhance the credibility and trustworthiness of the analysis, the remaining members of the research team reviewed the final thematic structure and qualitative data reporting in the form of a written report, providing validation and critical feedback. This process ensured a rigorous and reflexive approach to theme development, grounded in collective interpretation and consensus building amongst researchers and practitioners across the team.

In addition, team reflections were discussed weekly and recorded in a shared file. Authors contributed variably to the project stages; some completed the questionnaire and others focused on data analysis and writing.

## Main discussion

The main section of this commentary will delve into four critical areas: ethics and governance, collaborative working, team communication, and information sharing. These four critical areas emerged organically as the project progressed, through ongoing team reflections, weekly meetings, and questionnaire feedback, which collectively highlighted the most persistent and impactful challenges experienced across the partnership These reflections were noted in a shared team document on an ongoing basis and were often discussed in the weekly project meetings. These reflections are supplemented by insights from an online questionnaire conducted among the partnership team.

### Ethics and governance

Navigating the research ethics and governance landscape presented significant challenges within our collaborative partnership, as highlighted by the questionnaire data. The partnership involved diverse organisations, each with its own set of ethical guidelines and governance frameworks, whilst also being situated within the Health Research Authority remit given its focus on health and social care research and involving a vulnerable population. This diversity required careful alignment to ensure compliance across all organisations to their particular individual ethical governance procedures (where relevant), which the participants (project team) found to be a complicated and frustrating process.

From a total of 9 completing the questionnaire, 5 of 9 respondents were unsure if the project’s primary goal—establishing a new working partnership—was achieved. With just under half of the respondents being unclear whether the working partnership had been established on the questionnaire, we analysed the earlier reflections to understand why this may be the case. The reflective notes suggested that this level of uncertainty was in part due to ethical and governance challenges earlier reflected upon in project meetings. Reflections included noting where delays and tensions arose due to differing organisational priorities and differences in Standard Operating Procedures around ethics and governance processes. Additionally, the qualitative data in the questionnaire responses further supports these earlier reflections, with participants describing the ethical processes as incompatible with, in particular, the VCSE context. One participant noted:

*“Trying to fit a complex research project requiring strict ethical and governance approvals into a community organisation has not been fit for purpose”.*

Another central issue was the appropriateness of the Health Research Authority (HRA) ethics processes, which we perceive to be primarily designed for Randomized Controlled Trials (RCTs) and Clinical Trials of Investigational Medicinal Products (CTIMPs). These processes are often ill-suited for the type of community-based research such as the research undertaken in this project, leading to significant delays and complications. For example, the HRA required documents such as a Schedule of Events, whilst the specific NHS Trust’s Capacity and Capability assessment documents were originally designed for RCTs and CTIMPs, making them largely irrelevant to this project’s design and context. The difficulty of ‘translating’ the documents for a non-RCT study had several implications. The first being that the terminology itself was a barrier to the VCSE organisation and community engagement in this project. Additionally, this terminology was not wholly appropriate to our research design, e.g. qualitative research, further adding to the confusion and complexity in such a research context and partnership. Secondly, we perceived the level of ethical documentation to be overly complex and restrictive in terms of how you report study designs that are non-RCTs proportionate to the level of risk within our study and studies similar to ours. Whilst we fully and whole-heartedly support rigorous ethical processes and procedures, we do champion use of Plain English to increase accessibility and translation of research. This could foster tensions between the need for legal precision and the requirement for accessibility.

Complexity was further compounded by additional governance requirements, such as the need for the National Institute for Health and Care Research (NIHR) Good Clinical Practice (GCP) training. GCP training was asked of all project members, however, in reality such training was irrelevant to many of the partnership members in this research context; namely because not everyone had direct (or any) contact with the research participants and GCP training often goes into a lot of detail that wasn’t required for our study design (e.g. CTIMPS). Added to the challenges surrounding training, was the lengthy process of obtaining Letters of Access for community partners and academics to work with individuals living with SMI, which added unnecessary complexity. Furthermore, to obtain these Letters of Access there was a requirement to have occupational health clearance. This proved impossible within our partnership context, as the VCSE organisation (a charity) did not have an occupational health or human resources team. This meant that occupational health clearance, including the need for vaccinations, proved a difficult process to navigate. Negotiations were required with the sponsor (being TEWV NHS FT, who took on legal and ethical responsibility for the research) to mitigate the need for hepatitis B vaccinations, and the sponsor had to undertake occupational health screens for the VCSE organisation staff.

Moreover, the requirement to seek amendments to the approved ethics applications as the project evolved in a dynamic research environment also created substantial delays, even when the amendments pertained to relatively minor concerns. These delays frustrated the project team, particularly VCSE partners, as they hindered progress and exacerbated existing tensions between legal governance needs and the practical demands of community-based research. This is directly relevant to tensions described already in the literature around the length of time it takes for research to have meaningful impact [26]. These challenges also underline the need for a more flexible and context-sensitive research governance framework that can accommodate the unique needs of diverse research partnerships, particularly those involving community-based organisations, where practical matters will commonly supersede adherence to predefined research design protocols. Without such flexibility, the risk of delays and misalignments between partner organisations remains high, potentially undermining the effectiveness of collaborative research projects and leading to project delays, lack of community involvement in research, or even project failure.

### Collaborative working

The collaborative nature of the project was both a strength and a challenge. The quantitative questionnaire data indicated mixed feelings about the overall experience, with only 3 of 9 participants rating collaboration as positive, while the majority (*n* = 6) of participants had mixed experiences. These quantitative findings reflects the qualitative questionnaire feedback, where participants expressed satisfaction with the project’s impact despite the challenges. One participant noted:

*“The benefit for co-delivering a mixed-method project far outweighs the challenges*,*”*

emphasising the value of interdisciplinary collaboration despite the hurdles.

Furthermore, one of the primary challenges was the difficulty in aligning each organisational culture to ensure smooth project development and completion. Organisational cultures include specific information and communication technology requirements, specific demands on data management, differing interpretations of organisational hierarchies (e.g. the role of the Principal Investigator and what that means in different organisations) and so on. Each of these areas differed across the partners, meaning that those with the most experience of working across partner organisations (i.e. those with University and NHS experience) experienced far higher workloads to complete the “boundary work” [27] of translating organisational cultures across the team. This additional workload led to enhanced pressure for those involved. We assert that planning out a shared language of roles and responsibilities and making time to communicate differing interpretations of common research frameworks from the start is vital to project success. However, it is also important that organisational cultures become flexible enough to adapt as the project progresses and evolves.

Our questionnaire results also reflected these collaboration difficulties, with 6 of 9 participants indicating that project challenges were not always appropriately addressed in a timely manner. This suggests that, whilst a collaborative approach enriched the research, it also required more structured support– and time - to manage the complexities inherent in such partnerships. Therefore, funders may need to be aware of such rigidity in existing frameworks and differing organisational cultures and allow for longer time periods in which to conduct such research. This would help to allow sufficient time to navigate within and around research systems for the best outcome for all involved and the research itself.

The project also faced difficulties related to project governance, given that the leadership involved two joint Principal Investigators (PIs), and Co-Investigators (CIs) and researchers from different organisations. This sometimes made it challenging to collaborate, including who should delegate and divide tasks. This situation was further complicated by the diverse legal, HR, and organisational structures across the partner institutions, making it difficult to align processes and expectations. Additionally, whilst the VCSE organisation CI received the largest Full Time Equivalent (FTE) allocation for overseeing the project, in reality, VCSE organisations are often working in challenging contexts themselves, meaning that more urgent priorities take precedence. This, in our project, meant the second NHS-based CI had to deliver on most of the project oversight within a more limited FTE allocation, thus contributing to pressures. There are many implications arising from this. One implication is for research funders to ensure focused and tailored programme support is in place for new CI’s (particularly from VCSE organisations), with regular check-ins to ensure deliverability of the project. This is starting to happen in some contexts, such as Research Support Services for Public Health Research [28], but may not be as available within other contexts and research disciplines. A second implication is to consider the CIs having the same FTE allocation to ensure sufficient time for oversight, mentorship and training. This is often tricky within budgetary parameters.

The absence of earlier meetings and discussions within the project delivery team likely contributed to these challenges, particularly in clarifying roles, responsibilities, and integrating lived experience into the research process. While such conversations could have alleviated some issues, the tight timeframes made it difficult to prioritise and allocate sufficient time and support to these tasks. Participants noted that such early engagement would have provided a better understanding of the project’s overall aims and each member’s role, potentially preventing some of the miscommunications and inefficiencies that arose later.

In summary, while the collaborative approach enriched the research, it also required more structured support to manage the complexities inherent in such partnerships. The differences in organisational cultures, communication styles, and operational expectations were significant challenges that needed to be addressed proactively to ensure smoother collaboration and more effective project outcomes. Overall, while the collaboration enriched the research, it required structured support and proactive measures to address significant organisational, communicative, and operational differences. Establishing clear communication channels, workload management strategies, and training on organisational processes early on can facilitate smoother collaboration and improve project outcomes.

### Team communication

Effective communication was identified as a cornerstone of the project’s success, yet it was also one of the most challenging areas within the project delivery. The quantitative questionnaire data showed that team communication was generally rated positively, with a median score of 4 out of 5. This suggests that many partners perceived communication within the team as good. However, this overall positive rating masks underlying issues that were highlighted in the qualitative feedback, where more nuanced problems in communication were frequently discussed.

Participants repeatedly mentioned that communication breakdowns were a significant challenge, particularly due to the project’s dual leadership structure. This structure, intended to bring together expertise from different organisations and to upskill the VCSE, instead sometimes created confusion and inefficiencies. One participant noted, *“Lack of clarity from one PI regarding the delegation of tasks and duties…created tension within the team*,*”* highlighting how the ambiguity in leadership roles led to miscommunication and delays. The dual leadership model, without clearly defined responsibilities and communication channels, often left team members uncertain on who was responsible for what, which in turn hindered the smooth progression of the project.

Additionally, communication gaps during key decision-making stages were particularly problematic. These gaps often resulted in miscommunication or duplication of efforts, where different parts of the team might have been working on similar tasks without realising it, or where decisions were made without fully informing all stakeholders. This lack of synchronisation not only slowed down the project but also led to frustration among team members, as it sometimes felt that their efforts were being wasted or not fully utilised within a large partnership team.

These issues were further exacerbated by the differences in organisational cultures and expectations. The project brought together partners from various sectors, including the NHS, academia, and the voluntary and community sectors. Each of these organisations had its own norms, communication styles, and expectations about how work should be conducted. These cultural differences sometimes led to misunderstandings, where actions or decisions made in one part of the team were not properly communicated or were misunderstood by others. Compounding this were issues around shared communication and data management channels, whereby each organisation had different requirements for which platform to use, which not everyone had access to. What may be a seemingly small issue, therefore proved challenging when trying to communicate and share documents with many team members and across organisational boundaries.

The questionnaire data also revealed that only 3 of 9 participants believed that these communication challenges were always appropriately handled. This low number indicates that many team members felt that communication lapses significantly impacted the project’s ability to address issues in a timely and effective manner. When challenges arose, the lack of clear, consistent communication often meant that problems were not addressed as quickly or as effectively as they could have been, leading to further delays and complications.

To address these recurring communication challenges, future projects would benefit from an initial focus on establishing clear communication protocols tailored to multi-institutional teams. Developing an agreed-upon framework with well-defined roles, decision-making processes, and preferred communication tools could foster smoother collaboration. Additionally, providing training on each organisation’s communication practices and expectations early in the project could help bridge cultural differences, enhancing team members’ understanding of each other’s operational styles and preventing misunderstandings. These measures could ensure that future collaborations avoid common pitfalls and operate more seamlessly across diverse organisational boundaries.

### Data protection and sharing

The utilisation of online communication and data-sharing platforms has become a standard practice across multiple sectors, particularly within academic settings. Our project partnership primarily intended to employ Microsoft Teams and Sharepoint as the principal communication and collaboration tool, facilitating easy file access across the partnership and real-time document editing. However, this encountered several issues, which adversely impacted the project’s overall efficiency.

The primary issue was related to document sharing due to compliance with data protection regulations. In the UK, the use of cloud-based services such as Microsoft Teams within an organisation is predominantly governed by compliance requirements to legislation. Such legislation includes the General Data Protection Regulation (GDPR), supplemented by the Data Protection Act 2018 [29]. These regulations stipulate that the personal data of EU/UK citizens must be stored within the EU/UK or in countries that provide an adequate level of data protection [29]. Conversely, cloud-based data sharing platforms such as Microsoft Teams, SharePoint and OneDrive often have servers located across the globe, some of which might not be considered as having an adequate level of data protection by the UK’s authority. In practice, organisations such as the university partner in this project often have bespoke agreements with specific cloud-based service providers, such as agreeing on specific data storage locations to enable them to use these services. These locations are not restricted to the UK. However, for NHS trusts, all NHS data must be stored in the UK and be encrypted using specific methods [30]. This restriction applies to all data, not just personal data, whereas GPDR and DPA2018 implication applies to personal data only. This conflict prevented the partnership from using Microsoft Teams as cloud-based file-sharing platform for all project files, including those that do not contain personal information such as project planning, task lists, and Gantt charts. Similar challenges were also discussed in medical research, specifically when the partnership involves members from different countries [31].

As a compromise, the partnership decided to utilise the university partner’s proprietary shared drive with servers located within the university. However, this led to other issues such as reduced accessibility compared to Microsoft Teams due to the requirement of a university IT account for access, particularly affecting project members from the community organisation.

Participants expressed frustration over this issue. One participant noted:

*“Not being able to store project documents on Teams was a real challenge. It meant non-university partners could not access up-to-date information and also stopped multiple users being able to edit at the same time”.*

This limitation significantly reduced the efficiency of the teamwork and often resulted in team members duplicating efforts on tasks already completed by others. This most likely also contributed to a feeling of communication breakdown.

### Operational complexity in co-produced, community-based research

Many of the challenges outlined above impacted on the research project delivery. Within our partnership context, challenges included the use of surplus food as ingredients in the ready meals. For clarity, surplus food is defined as food that is produced or available in quantities exceeding demand. Such surpluses arise from inefficiencies in the food supply chain, including agricultural overproduction, manufacturing scheduling errors, retailer overstock, strict cosmetic standards, and market fluctuations. One of the aims of our project was to design and produce food products utilising surplus food to reduce waste. However, practical implementation revealed several challenges, again highlighting the complexity of community based, co-produced research.

As an example of delivering complex research project outcomes, one of our challenges was the inherent unpredictability of surplus food supplies, particularly within research projects which have fixed timelines. This unpredictability complicated both the recipe development and production stages of our research. Due to requirement of the food safety legislation, a bespoke production process adhering to Hazard Analysis Critical Control Point (HACCP) principles needed to be designed for each product. This resulted in several weeks’ lag between the finalisation of the recipe and the dates of the scheduled production. Although long shelf-life food items can be stocked, fresh ingredients must be sourced on the production day, significantly limiting the project’s production scale. Our earlier work with Fare Share North East, a UK charity that redistributes surplus food from the food industry to nearly 8,500 charities and community groups, suggested that there was potential to identify patterns of supply availability. Surplus supply is likely to exhibit seasonal patterns, particularly for fresh produce. For example, pumpkins are reasonably expected to be available after Hallowe’en (31st October) in the UK. Different modelling techniques have been applied to explore the patterns of food donation and distribution for a large food rescue organisation in Australia, demonstrating this is possible with enough data [32].

These variations led to operational challenges. For example, the irregularity and seasonal variability of surplus food supplies which necessitated deviations from the original project protocols, required the team to adapt recipes and production schedules in real-time, which initially posed some difficulties. This contributed further to project team stress and delayed the project timeline. These challenges illustrate the specific regulatory, temporal, and logistical difficulties that can arise when translating co-produced ideas into practice, particularly within interdisciplinary collaborations that bring together sectors with different norms, requirements, and paces of work. In this case, the intersection of health research, food safety regulation, food logistics, and community engagement revealed tensions that required continuous negotiation and adaptation.

Thus, there is a significant need for funders, particularly those traditionally operating in health research, to recognise and be flexible in order to accommodate necessary deviations from the initial protocol in multidisciplinary project teams. More broadly, this highlights the importance of embedding operational flexibility within interdisciplinary co-production frameworks, especially where policy, regulation, or supply chains are involved, as challenges in one domain (e.g., food logistics) can significantly impact other domains (e.g., research ethics or timelines).

Implications for future community collaborative work include allowing sufficient time to access many different forms of data, for example, the surplus food provider’s inventory data to identify supply patterns and predict food ingredient availability throughout the year. This approach would then enable the creation of recipes, development of products and HACCP plans, and scheduling of production in advance to utilise these materials when they become available. The second implication for such complex research might also be a need to bring on-board even more project partners, further adding to the complexity. In our case, this would be a surplus food supplier as a project partner (such as Fare Share North East), which would allow us to access their supply data for pattern analysis and real-time supply information. Further growth in the number of partners would therefore potentially necessitate more clarity in all of the above aspects, such as funding envelopes, timescales, communication channels and ways of collaborative working. From a broader perspective, the management of such complexity offers transferable insights for other co-production initiatives, particularly those situated in cross-sectoral settings where regulation, logistics, and community expectations must be aligned. Following the learning from this project, we are carrying out further works, partnering with Fare Share North East to address these logistic and operational challenges.

## Summary/key conclusions

Through this complex, inter-disciplinary, community-based health research project, we hope that our experiences highlighted above, from the integration of questionnaire data and reflections, may illustrate the complexities in such research and offer careful thinking points for others wishing to work in similar contexts. It also suggests more systemic, structural adaptations to the research landscape, for which a dialogue needs opening. In our specific case, whilst our project achieved several positive outcomes, particularly in fostering interdisciplinary collaboration and producing meaningful research results, it also highlighted significant challenges in the areas of ethics and governance, collaborative working, and team communication. Based on these challenges, it is clear that funders and research leaders need to adopt more flexible and proactive approaches to managing multi-partner collaborations, and consider the wider research landscape, frameworks, infrastructures, procedures and expectations that currently exist.

Compared to existing studies [33–35] of co-production, this project highlights specific challenges around integrating operational constraints from sectors like food redistribution into health-focused research. The role of surplus food logistics is rarely discussed in the co-production literature [36] and this project provides unique insight into adapting governance, communication, and delivery frameworks to meet both health and sustainability goals.

Navigating ethics and governance frameworks presented significant challenges due to differing organisational priorities and processes, particularly between healthcare and community-based research. However, these challenges point to several potential solutions that can improve future collaborative research. First, establishing a flexible ethics framework that accommodates community-based research could significantly reduce delays. This would involve creating tailored ethical guidelines that address the specific needs of non-clinical research contexts, such as those encountered in the voluntary sector. Streamlining processes, such as simplifying documentation for community partners, would also be beneficial. For instance, shortening approval processes for projects that do not require clinical trials can help alleviate this burden. Additionally, enhancing communication between ethics boards and research teams early on could resolve issues before they cause delays. Providing training sessions on how to navigate ethics processes for all partners, including those unfamiliar with healthcare-based governance, would ensure better alignment. Generic ethics training is provided by many Universities in the UK, however, the level to which it meets the needs of different types of organisations (i.e. non-HEIs) is uncertain.

While the interdisciplinary nature of the project brought invaluable perspectives, challenges arose from uneven task delegation and differences in organisational culture. To address this, future projects should implement clear workload management strategies from the outset. For instance, a project manager could be appointed to oversee task distribution and ensure that all partners, particularly those with heavy responsibilities, are not overburdened. The project manager should maintain ongoing communication with project partners, as the workload for each partner will vary throughout the project’s duration. Furthermore, early cross-organisational training on communication and IT requirements could help bridge the gaps in understanding different working processes. This would foster greater synergy among partners by developing a shared understanding of each organisation’s methods and expectations.

Effective communication, though rated positively by the majority of participants, suffered from ambiguities in leadership and decision-making processes. A clear solution is the implementation of a leadership structure with defined roles and communication protocols. Establishing formal communication channels, where responsibilities are clearly described, would ensure that decisions are communicated effectively across all levels of the team. Additionally, periodic check-ins to reassess communication efficiency and address emerging challenges would further enhance team coherence, preventing the lapses that led to delays in this project.

The restrictions around using Microsoft Teams for document sharing, due to the variation of data protection requirements for different institutions, significantly hindered collaboration with the NHS deeming this platform non-compliant with their data protection requirements. To overcome this, future partnerships could explore secure cloud-based solutions that meet the stringent data protection needs of healthcare organisations while allowing real-time collaboration. This might involve negotiating bespoke agreements with cloud service providers to guarantee compliance with regulations such as GDPR. In the interim, creating a hybrid system combining secure cloud storage for sensitive data with universally accessible collaboration platforms for non-sensitive materials could strike a balance between security and functionality. Overall, whilst for us, this project has had several challenges, these learning points have been invaluable. It has allowed us to plan for the follow-on research being much more mindful of such challenges and has hopefully opened up a debate around flexibility in the system. We hope these reflections are useful for others working in other disciplines and research areas, but which are complex inter-disciplinary projects.

In conclusion, while cross-sector collaborations offer significant potential for innovation and social impact, they also come with inherent challenges that must be addressed proactively. By learning from the difficulties encountered in this project—ranging from ethical misalignments to communication breakdowns and data-sharing issues—future partnerships can be better equipped to manage the complexities of multi-organisational collaboration. Implementing flexible governance frameworks, improving communication protocols, and ensuring better resource management will not only enhance the efficiency of these projects but also increase their likelihood of success. Funders, in particular, must recognise the need for adaptability and flexibility within project timelines and expectations, allowing for necessary deviations in response to the practical realities of collaborative research.

### Strengths and limitations

This project demonstrates the potential of co-produced, community-based research in addressing both nutritional and operational challenges. A key strength was the interdisciplinary team and inclusion of a VCSE partner, which enabled real-world insights. However, the small number of questionnaire respondents (*n* = 9), all internal to the team, limits the generalisability. Transferability is also constrained by the localised nature of the food redistribution system. Nonetheless, the lessons learned offer valuable insights for similar interdisciplinary and cross-sector collaborations.

## Supplementary Information

Below is the link to the electronic supplementary material.

Supplementary Material 1

## Data Availability

The datasets used and/or analysed during the current study are available from the corresponding author on reasonable request.
