# Supplementary material for: Square peg round hole: navigating operational and interdisciplinary challenges in a co-produced, community-based research partnership
Source: Res Involv Engagem. 2025 Aug 18;11:98. doi: 10.1186/s40900-025-00769-1 (PMC12362993; doi:10.1186/s40900-025-00769-1)
Supplement: Supplementary file 1 — Supplementary Material 1 [file 40900_2025_769_MOESM1_ESM.docx]

**Increasing accessibility of affordable healthy food to adults living with Severe Mental Illness in Middlesbrough**

**Survey Questions (including consent)**

**INFORMED CONSENT (participants will be unable to continue unless all boxes are ticked)**

Thank you for agreeing to take part in this survey. Before continuing to the survey questions please initial the following statements to show that you agree and are happy to continue.

|  | I confirm that I have read the Participant Information Sheet [INSERT VERSION NUMBER AND DATE]. |
| --- | --- |
|  |  |
|  | I understand that my participation is voluntary, that I can choose not to participate in part or all of the survey. I can withdraw from the survey up to the point of submitting the survey, without giving a reason. However, if I submit the survey, I will not be able to withdraw. |
|  |  |
|  | I understand that non-identifiable research data will be stored indefinitely on a secure password protected server at Teesside University and the non-person identifiable research dataset may be made publicly available (for example, as a supplement to the journal article, or stored on an on-line scientific data repository). All information will be treated as confidential in accordance with the General Data Protection Regulation and the Data Protection Act 2018. |
|  |  |
|  | I agree that anonymous data can be used to write up research reports, journal articles, conference presentations or any other dissemination activity. |
|  |  |
|  | I agree to take part in this study. |

**ONLINE SURVEY**

This survey is designed to evaluate people’s experience of working on this study. It is broken down into three sections: Questions about you and your place of work; questions about your experience of working on the project; and questions about future work and recommendations for future research partnerships.

There are no right or wrong answers, and you do not have to share anything that you feel uncomfortable with. Please be as open and honest as you feel able and offer detailed answers where you feel you are able.

##

## **Questions about me and my place of work**

**1. Have you ever worked on a funded research project before (excluding any research done as part of a qualification e.g. Undergraduate or Masters degree, or a PhD)?**

Yes

No

Prefer not to say

**2. How many years have you been working on funded research projects (not including studies for your qualification)?**

Free text answer.

**3. Have you worked with NHS patient-focussed research before (i.e. research that requires ethical approval from a Research Ethics Committee)?**

Yes

No

Don’t know

Prefer not to say

**4. Which organisation are you primarily employed by?**

Tees, Esk and Wear Valleys NHS Foundation Trust

Middlesbrough Environment City

Teesside University

Other (please state)

Prefer not to say

**5. Have you participated in a new collaborative partnership before for example between the NHS, universities, social care, and/or third-party organisations?**

Yes

No

Don’t know

Prefer not to say

## **B. MY EXPerience of working on this project**

**6a. How would you describe your overall experience being involved in this project?**

Positive

Negative

A mixture of both

Prefer not to say

**6b. Please give your reasons for giving this overall rating of your experience, in your own words.**

Free text answer

**7.Do you feel that you had enough time allocated to work on this project?**

Yes (please go to question 9)

No (please go question 8)

Don’t know (please go to question 9)

Prefer not to say (please go to question 9)

**8.How much time do you feel would have been more appropriate to complete all of the duties allocated to you on this project?**

Free text answer

**9.Do you feel you had sufficient training to undertake your role on this project?**

Yes (please go to question 11)

No (please go question 10)

Don’t know (please go to question 11)

Prefer not to say (please go to question 11)

**10. If you felt you needed more training to carry out your duties, please explain what training would have helped you.**

Free text answer.

**11. Did you fully understand the project and what was required from you in your role?**

Yes (please go to question 13)

No (please go question 12)

Don’t know (please go to question 13)

Prefer not to say (please go to question 13)

**12. How could the team have helped you understand the project and your role better, and who could have helped explain this to you?**

Free text answer.

**14. Do you feel that the overall goal of the project, to establish a new working partnership, has been achieved?**

Yes

No

Don’t know

Prefer not to say

**15. Please briefly explain your reason for your answer to the previous question about the goal of the project.**

Free text answer.

**16. How often did you experience challenges/ unexpected circumstances in this project?**

Frequently

Occasionally

Never

Don’t know

Prefer not to say

**17. Please briefly outline any challenges or unexpected circumstances you experienced during the project.**

Free text answer.

**18. Do you feel that the processes followed throughout this research were all required (such as ethics approval, writing protocols and reporting recruitment data)?**

Yes (please go to question 20)

No (please go to question 19)

Don’t know (please go to question 20)

Prefer not to say (please go to question 20)

**19. Please briefly outline which processes you felt were not necessary and why.**

Free text answer.

**20. Do you feel like each partner brought strengths/resources/contacts to the project that would not have been there without them?**

Yes

No

Don’t know

Prefer not to say

**21.In your opinion, were all challenges/barriers dealt with appropriately and in a timely manner?**

Always (please go to question 23)

Sometimes (please go to question 22)

Never (please go to question 22)

Prefer not to say (please go to question 22)

**22. What could have been done differently to overcome the challenges and barriers that arose during this project in a more timely or appropriate manner?**

Free text answer

**23. Please rate the following aspects of the project from 1-5, with 1 being extremely poor; 2 poor; 3 satisfactory; 4 good; and 5 excellent.**

|  | 1 | 2 | 3 | 4 | 5 |
| --- | --- | --- | --- | --- | --- |
| Team dynamics |  |  |  |  |  |
| Team communication |  |  |  |  |  |
| Team leadership |  |  |  |  |  |
| Team collaboration |  |  |  |  |  |
| Team support available |  |  |  |  |  |
| Day to day working |  |  |  |  |  |

**24. Is there anything else you would like to tell us about your experience of working on this project or suggest to help improve future projects like this one?**

Free text answer.

## **C. Future Work and recommendations**

**25. Would you be happy to work in this partnership again?**

Yes

No

Don’t know

Prefer not to say

**26. How do you feel that this study could influence future studies involving research partnerships?**

Free text answer.

**27. Finally, do you have any additional comments or recommendations for future researchers working on a collaborative project like this one?**

Free text answer.

**FINAL PAGE:**

Thank you for taking the time to complete this survey. You may find writing about your experiences of the project upsetting. If you feel distressed, you can contact the Samaritans on 0330 0945717 or you can contact the Crisis Team at Tees, Esk and Wear Valleys NHS Foundation Trust on 0800 0516171. Both lines are open 24 hours a day, 7 days a week to support people in distress.
